# Recent advances in liver transplantation with HCV seropositive donors

**DOI:** 10.12688/f1000research.20387.1

**Published:** 2019-12-30

**Authors:** Soumya Murag, Brittany B. Dennis, Donghee Kim, Aijaz Ahmed, George Cholankeril

**Affiliations:** 1 Department of Medicine, Santa Clara Valley Medical Center, Santa Clara, CA, USA; 2 Division of Gastroenterology and Hepatology, Stanford University School of Medicine, Stanford, CA, USA; 3 Department of Health Research and Policy, Division of Epidemiology, Stanford University School of Medicine, Stanford, CA, USA

**Keywords:** HCV donor, liver transplantation, DAA therapy, hepatitis C virus

## Abstract

The paradigm shift from interferon-based to direct-acting antiviral (DAA) therapy for the treatment of hepatitis C virus (HCV) infection has revolutionized the field of liver transplantation. These advances in effective HCV treatment, along with the persistent shortage in available liver grafts, have encouraged investigators to assess the need for adopting more inclusive donor policies. Owing to the poor outcomes following liver transplantation with recurrent HCV infection, liver transplantation using HCV seropositive donors (non-viremic and viremic) had been restricted. However, as a result of the growing supply of HCV seropositive donors from the recent opioid epidemic along with the advent of efficacious DAA therapy to treat HCV recurrence, there has been an increasing trend to use HCV seropositive donors for both HCV seropositive and seronegative recipients. The review aims to discuss recent advances and associated outcomes related to the use of HCV seropositive grafts for liver transplantation.

## Introduction

As the number of registrants awaiting liver transplantation (LT) far exceed the supply of available liver grafts ^1^, there has been a greater focus on how to effectively use the available organs. Historically, donor liver grafts infected with hepatitis C virus (HCV) have been discarded or were used primarily in HCV seropositive recipients (viremic and non-viremic) because immediate and universal post-transplant HCV recurrence led to early allograft dysfunction in a significant proportion of recipients ^2, 3^. However, the advent of direct-acting antiviral (DAA) agents, especially with their favorable safety profile and efficacy, has greatly impacted the landscape for LT ^4^. For over two decades, HCV was the most common indication for LT in the US. However, since the US Food and Drug Administration’s approval of second-generation DAA agents in late 2013, the number of waitlist registrants and LT recipients with HCV has declined markedly. Despite the reduction in the waitlist burden related to HCV, the number of total registrants awaiting LT continues to rise. In 2016, for the first time, both alcoholic liver disease and non-alcoholic steatohepatitis (NASH) had surpassed HCV as the leading indications of LT in the US ^5^.

With early data demonstrating favorable outcomes with pre-emptive DAA treatment for HCV infection in the post-transplant setting, the use of HCV seropositive donor liver grafts has increased because of the growing shortage of available donor organs ^6^. Moreover, in the setting of the recent opioid epidemic in the US, there is an increased prevalence of young, otherwise-healthy, HCV-infected potential donors ^7, 8^. With the combination of the shortage of organs along with the changing landscape of HCV treatment with DAA agents and the opioid epidemic, it is an opportune time to expand the donor pool and improve overall outcomes for registrants awaiting LT. This review aims to assess the advances in the use of HCV seropositive donors which includes both HCV non-viremic (RNA-negative) and HCV viremic (RNA-positive) donor liver grafts in HCV seronegative recipients ^9^.

## Current trends in the demand for liver grafts

Amidst an ever-increasing shortage in donor organs, several policy changes have been implemented that prioritize medical urgency of LT rather than geography. For example, the search for a better allocation system led to the adoption and implementation of the Model for End-Stage Liver Disease (MELD) score which is composed of objective parameters that reflect medical acuity and necessity for LT. Over the past two decades, additional exception policies to the MELD score have been used to further prioritize medical urgency despite geographic location (Status 1A and Share 35 policies) as well as appropriately selecting and transplanting patients with hepatocellular carcinoma ^10^. This has led to a significant rise in the rate of LT surgeries performed, with a landmark 7841 LT surgeries in 2016 ^1, 10^, the majority of which were from deceased donors. Moreover, deceased donor LT outcomes have improved; the incidence of post-transplant mortality and graft failure at 1 year decreased to under 10% for recipients.

Despite these advances, there continues to be an increasing annual number of waitlist registrants added to the LT list with a decreased number of candidates waiting at the end of the year, indicating rising rates of LT surgeries per year ^1^. Projection models predict that the waitlist burden will continue to rise from the growing epidemic of NASH over the next two decades ^5^, and current statistics suggest that HCV is no longer the most common indication for transplantation.

Owing to this persistent shortage of livers, attention has been given to expanding donor transplantation criteria to increase the available donor supply. Initially, this included using organs previously thought to be high-risk, such as those organs donated after cardiac death, from advanced donor age, and with minimal hepatic steatosis ^11, 12^. Living donor LT also had a great impact in expanding the donor pool and significantly shortened waiting times for recipients. Among these expanded criteria, the use of HCV seropositive liver grafts has increased because of the availability of effective therapies to treat HCV (DAA agents).

Currently, there are no consensus guidelines on the threshold of underlying degree of fibrosis in procured HCV-positive grafts compared with non-HCV grafts in the DAA era. The general acceptable cutoff is less than stage 3 fibrosis (F3) ^10^. With pre-emptive DAA treatment in the peri-transplant setting, these concerns on fibrosis may be mitigated; however, further studies are needed to clarify this. Careful consideration should be taken when using HCV viremic or non-viremic liver grafts, including minimal steatosis (<30% hepatocytes), necrosis (<10%), and no more than mild non-specific portal inflammation on liver biopsy at the time of procurement ^10^.

Prior to the availability of DAA agents and during the interferon era, HCV seropositive donors were three times more likely to be discarded than HCV seronegative donors or not considered for procurement at all. Currently, the overall proportion of livers discarded decreased from over 20% in 2010 to 9.0% in 2016; this was likely a reflection of the dramatic decrease in discard rates of HCV seropositive non-viremic donors. The dramatic reduction in discard rates can be explained in part by the mandated testing for HCV RNA and the ability to identify non-viremic donors. As of 2016, HCV seropositive non-viremic and HCV-negative donors had similar discard rates (9.0% and 8.9%, respectively), a result of the effective and well-tolerated DAA agents ^1, 2^.

The outcomes after transplantation with HCV seropositive viremic and non-viremic donors in both HCV seropositive and HCV seronegative recipients are discussed below.

## Natural progression of HCV in the post-transplant setting

The discussion of antiviral therapy is important as it relates closely with the new trends in HCV donors in LT. HCV is a positive-stranded RNA virus, and testing of HCV includes serologic studies of anti-HCV antibody (HCV Ab), which indicates exposure to the infection and may take 6 to 8 weeks to appear, and nucleic acid testing (NAT), which confirms active HCV infection and is detectable within 2 weeks of exposure. The eclipse period between viral exposure and positive NAT result is 5 to 7 days, whereas the window period from exposure to positive antibody test is 60 to 70 days. This is particularly relevant in intravenous drug users who have the highest risk of negative test results in the eclipse period ^13^, and repeat NAT testing should be considered in grafts that were NAT-negative at the time of procurement among all donors with increased risk for potential transmission.

Previously, HCV-positive donors referred to anyone with positive HCV Ab; however, this is incomplete as it does not differentiate between the presence and absence of viremia. Therefore, the new consensus suggests that any HCV-positive donor refers to those with positive NAT results ^13^. The American Society of Transplantation Consensus Conference calls for a shift to replace the term “HCV‐positive donor” with the term “HCV‐viremic donor” ^13^.

The natural progression of untreated HCV infection in LT recipients is well known ^14, 15^. Those with HCV viremia prior to transplant inevitably experience HCV recurrence post-transplant, resulting in graft dysfunction and progression to cirrhosis ^14^.

Although DAA agents have been highly effective, little is known about the optimal time to provide HCV treatment of HCV-infected recipients—before versus after transplantation. Treatment for renal transplant recipients is similar with two main strategies: treating HCV-positive recipient either before or after transplantation. Treatment of HCV infection prior to kidney transplantation offered 0.43 more life-years than HCV treatment after transplantation; however, according to base-case analysis, transplantation of HCV-infected kidney first with HCV treatment performed after transplantation was preferred for organ utilization and cost-effectiveness ^16^. Similarly, a recent analysis has shown cost-effectiveness in treating HCV patients prior to transplantation if the risk of hepatic complications is modifiable with HCV treatment, reducing the cost burden from recurrent hospitalizations. However, treatment of HCV in those with advanced liver disease may not be cost-effective; while the MELD score may improve, ongoing poor health outcomes and repeated hospitalizations may not. After achieving viral clearance prior to transplantation, patients with HCV cannot receive livers from HCV-positive donors, significantly limiting access to LT. In addition, recipients treated for HCV prior to transplantation would need to be retreated after transplantation if receiving a liver from an HCV-positive donor, adding to the health-care cost. Therefore, the decision for HCV treatment prior to LT depends on the local and regional availability of HCV-positive donors. If there is a high proportion of HCV-positive donors, it may be beneficial to treat after transplantation.

Owing to the lack of large prospective data, there is no consensus on the timing of post-transplantation HCV treatment—early versus late treatment. Until 2011, HCV was treated primarily with a combination of pegylated interferon and ribavirin. However, their poor tolerance often led to necessary dose reductions or treatment discontinuations, along with sustained virologic response (SVR) of as low as 30.2% ^14^. Therefore, interferon therapy was used primarily for treatment of HCV in liver recipients after transplant only if fibrosis was seen on biopsy ^15^. However, with the advent of DAA agents, evidence favors treatment of HCV recurrence early in the post-transplant setting before the onset of fibrosis, leading to improved patient outcomes ^17^. One study compared three DAA strategies: prior to transplantation, at the time of transplantation, and then at disease recurrence ^18^. With the assumption of 96% probability of achieving SVR, DAA therapy remained the most cost-effective when used pre-transplantation in those with decompensated cirrhosis with a MELD score of less than 20. However, for a MELD score of more than 20 or for patients with HCV, treatment at the time of recurrence is most effective when compared with treatment at the time of transplantation and pre-transplantation ^18^.

## Treatment strategy in HCV recurrence post-transplantation

Treatment data of those with HCV recurrence in an HCV seropositive recipient after transplantation differ from those who are HCV seronegative recipients. The latter will be highlighted later in the discussion as most data are in non-LT patients. The emergence of second- and third-generation DAA agents has resulted in SVR rates as high as 100% when used during LT ^19– 21^. Several studies have attempted to demonstrate the efficacy of various treatment regiments for different genotypes of HCV.

In 2016, the American Association for the Study of Liver Disease (AASLD) and the Infectious Disease Society of America (IDSA) co-jointly published treatment guidelines for the recurrence of HCV infection after transplantation. Patients with chronic HCV received 12 weeks of sofosbuvir/velpatasvir after recurrence of HCV after transplantation and showed a 3-month follow-up of 96% cure ^22^. In patients with HCV genotype 2 or 3 infection, non-ribavirin-based formulations include a 12-week daily course of glecaprevir/pibrentasvir or sofosbuvir/velpatasvir for LT recipients with or without compensated cirrhosis ^23^. Its pangenotypic treatment profile and efficacy have favorable SVR in smaller single- and multi-center studies as well.

Several landmark DAA clinical trials for the treatment of HCV are outlined below. The SOLAR-1 trial in 2015 demonstrates that sofosbuvir, ledipasvir, and ribavirin for 12 to 24 weeks achieved an SVR of 96 to 98% for HCV genotype 1 and 4 in transplant recipients without cirrhosis, and SVR was lower for patients with decompensated cirrhosis ^24^. ASTRAL-1 tested for all genotypes except genotype 3, finding an overall SVR rate of 99% when using a sofosbuvir and velpatasvir combination treatment for 12 weeks ^25^; ASTRAL-3 looked particularly at genotype 3, which showed superior SVR than standard treatment (sofosbuvir and ribavirin). Multiple trials have evaluated daclatasavir and sofosbuvir with or without ribavirin for 12 weeks in LT recipients with advanced cirrhosis with a 91 to 95% SVR among different genotypes ^26^. Special populations such as severe chronic kidney disease (stage IV) or end-stage renal disease are important to highlight. Particularly, sofosbuvir-based regimens have been safe and effective in those with stage IV or V chronic kidney disease. The combination of elbasvir and grazoprevir has been recommended for genotypes 1 and 4 for 12 weeks, and glecaprevir and pibrentasvir for all genotypes for 8 to 16 weeks ^27^.

## Outcomes of HCV non-viremic and viremic donor liver grafts into HCV-positive recipients

As of 2017, 16.9% of HCV seropositive LT recipients received liver grafts from HCV viremic and non-viremic donors ^2^. The majority of initial studies were conducted before the DAA era, and reported results are for HCV “positive” donors without any delineation regarding viremia. Initially, using HCV seropositive donors for immunocompromised recipients created anticipation. Since 1992, the majority of the studies showed no difference between HCV seropositive grafts and HCV seronegative grafts in HCV seropositive recipients ^28^. However, these outcomes did not measure long-term outcomes and lacked histological data such as fibrosis, which would give more compelling evidence. A cohort study among five different US transplant centers from 2002 through 2007 showed that transplantation with HCV seropositive grafts was associated with a 58% increased risk of advanced fibrosis ^12^.

One study assessed the recipients of 32 HCV seropositive donor liver grafts, of whom 15 had HCV viremia ^29^. These data demonstrated no significant difference in patient or graft survival for HCV seropositive recipients who received an HCV seropositive liver graft regardless of viremia status. Similarly, a matched case-control analysis in the European registry showed no difference in patient and graft survival when the three groups of HCV seronegative, HCV viremic, and HCV non-viremic grafts were compared. Among the HCV seropositive group, viremic donors predispose recipients to recurrent HCV infection and subsequent development of fibrosis after transplantation more than non-viremic donors. It was also evident that there is a higher prevalence of fibrosis in the group of HCV viremic donors than HCV non-viremic donors, leading to the question of whether HCV viremia status is a confounding factor in the analysis ^30^. Owing to the risk of advanced fibrosis, the main risk factor for early hepatitis C recurrence, most studies recommend wary use of HCV RNA-positive donors in HCV seropositive patients.

## Outcomes of HCV non-viremic and viremic liver grafts into HCV seronegative recipients

The safety and efficacy of DAA therapy in HCV-infected recipients have resulted in consideration of transplantation of HCV seropositive grafts in HCV seronegative recipients followed by early post-transplant treatment. Data regarding the use of HCV seropositive donors in HCV seronegative recipients are limited. Whereas discard rates of HCV non-viremic livers have decreased, discard rates of livers from HCV viremic donors continue to remain higher than 30% ^31^. Post-transplant viremia is evident even with donors who are found to be HCV non-viremic at the time of procurement. A recent report of 26 HCV non-viremic donor liver grafts transplanted into HCV seronegative recipients was studied, and 3-month post-transplant follow-up revealed that HCV transmission, which was confirmed by HCV NAT, occurred in four (16%) out of 26 recipients ^32^. This study was conducted in donors who met high-risk criteria for infectious transmission and may fall in the eclipse period where viremia was not accurately detected by NAT assay. Cotter *et al.* collected data from the Scientific Registry of Transplant Recipients for all recipients who underwent LT from January 2008 through January 2018. Graft survival at 1 and 2 years following LT from HCV viremic donors was similar to that of HCV non-viremic donors ^9^. A recent systematic review of 15 studies with HCV-positive donors assesses for patient and graft survival in HCV-negative recipients ^33^. Six of these studies were from national LT registries from both the US and multicenter European databases (sample sizes ranged from 38 to 1930 patients) and showed no difference in graft or patient survival. Overall, the HCV serostatus of recipient, not of the donor graft, was an independent predictor of graft survival ^12, 29^.

A Markov modeling study conducted in the US by Chhatwal *et al.* showed that HCV seronegative patients have an increased life expectancy by accepting any liver graft, regardless of HCV status if the MELD score was greater than or equal to 20 ^34^. Candidates in United Network for Organ Sharing (UNOS) regions with longer waitlist time to transplant may also benefit from accepting HCV seropositive donors. In HCV seropositive donors who had previously achieved SVR, using HCV viremic grafts may be associated with lower SVR and additional treatment costs but has still shown to be cost-effective in patients awaiting LT particularly with MELD scores above 23 ^35^.

Recent studies have evaluated national trends on the use of HCV seropositive donors into HCV seronegative recipients, stratified by donor viremia ^31, 36^. In total, 355 HCV seropositive liver grafts have been transplanted into HCV seronegative recipients from April 2015 to December 2018 in the US. Since 2017, there was an increase in LT from HCV non-viremic liver grafts to HCV seronegative recipients ranging from 1 to 8 per month and LT from HCV viremic donors to HCV seronegative recipients from 1 to 12 per month.

Transplantation of HCV viremic livers into HCV seronegative patients with prophylactic DAA agents could improve patient survival, and benefits may outweigh the harm of introducing a viral infection, particularly among those in need of immediate transplantation. Few trials have studied the efficacy of DAA agents for LT from HCV viremic donors to unexposed recipients, and most trials have been conducted in non-LTs. In a single-center non-randomized trial, non-HCV-infected recipients were treated prophylactically with grazoprevir and elbasvir immediately before and after transplantation from HCV viremic donors, and no HCV RNA was detected in recipients 12 weeks after prophylactic treatment before or after renal transplantation ^37^. It is difficult to conclude whether this provides true prophylaxis or early treatment of HCV infection.

A single institution study was conducted on adult HCV seropositive recipients who contracted HCV viremia after transplantation from HCV viremic donors. All patients began DAA therapy (sofosbuvir/velpatasvir/ribavarin combination) within 3 months of transplant and achieved SVR with no development of complications such as graft failure, fibrosing cholestatic hepatitis, or death ^38^. DAA therapy may interact with immunosuppressive drugs as the rate of biopsy-proven rejection was higher than the average rate seen in rejection of general LT. In fact, HCV treatment post-transplant has been associated with rejection and immune graft dysfunction.

## Conclusions

The recent opioid epidemic has resulted in a rising number of deaths among young, otherwise-healthy adults with HCV infection, contributing to the surge in available and used HCV viremic donors with favorable donor criteria. In addition, the era of DAA agents has seen a surge in the use of HCV-infected non-viremic and viremic donors in HCV-unexposed patients with early favorable outcomes. Overall, the outcome of HCV seropositive recipients who receive HCV viremic organs is limited with only a few liver, kidney, and heart transplants studied thus far.

The inequity in access to LT across the country contributes to the high waitlist death rate. Several questions remain regarding the accessibility to HCV treatment, and long-term outcomes need to be addressed in the near future.

## Abbreviations

DAA, direct-acting antiviral; HCV, hepatitis C virus; LT, liver transplantation; MELD, Model for End-Stage Liver Disease; NASH, nonalcoholic steatohepatitis; NAT, nucleic acid testing; SVR, sustained virologic response
